# Enhancing lateral flow immunoassay performance for cardiac troponin I detection with pore-size tailored silica nanoparticles and smartphone-based “AdaptiScan” analysis

**DOI:** 10.3389/fbioe.2025.1568719

**Published:** 2025-03-26

**Authors:** Shaonian Ye, Cifu Xu, Huilin Li, Shilun Feng, Yan Wang, Fang Gao

**Affiliations:** ^1^ Institute of Energy Materials Science, University of Shanghai for Science and Technology, Shanghai, China; ^2^ College of Information and Electrical Engineering, China Agricultural University, Beijing, China; ^3^ Department of Nephrology, Gongli Hospital of Shanghai Pudong New Area, Shanghai, China; ^4^ State Key Laboratory of Transducer Technology, Shanghai Institute of Microsystem and Information Technology, Chinese Academy of Sciences, Shanghai, China

**Keywords:** mesoporous, pore size, quantum dots, lateral flow immunoassay, smartphone-based analysis

## Abstract

The accurate and rapid detection of cardiac troponin I (cTnI) at the point of care is crucial for the timely diagnosis of myocardial infarction (MI). This study introduces an advanced lateral flow immunoassay (LFIA) platform for cTnI detection. We employed small-sized, large-pore dendritic mesoporous silica nanoparticles (DMSN-2) to encapsulate quantum dots (QDs), achieving an enhanced QD loading capacity of 1.427 g QD/g silica, compared to 0.881 g QD/g silica for smaller pore counterparts (DMSN-1). This nano-LFIA was further integrated with “AdaptiScan”, a smartphone-based detection system that uses adaptive detection algorithms to automatically extract and analyze fluorescence signals from LFIA strips. This integration of pore-size tailored DMSNs and “AdaptiScan” resulted in a limit of detection for cTnI of 42.6 ng/L, which meets clinical diagnostic requirements. The platform offers a sensitive, cost-effective, and portable solution for rapid detection of MI, potentially transforming point-of-care testing in resource-limited settings.

## 1 Introduction

Cardiovascular diseases (CVDs) remain the leading cause of global mortality, accounting for an estimated 17.9 million deaths each year, with myocardial infarction (MI) being a predominant contributor due to acute myocardial ischemia and necrosis (Vaduganathan et al., 2022). The cornerstone of MI diagnosis involves electrocardiography and the quantification of cardiac biomarkers, specifically cardiac troponin (cTn) (Thygesen et al., 2019; Byrne et al., 2024), which are present at extremely low levels (2–5 ng/L) in healthy individuals (Clerico et al., 2023). High-sensitivity cardiac troponin (hs-cTn) assays, with a limit of detection (LoD) ≤3 ng/L (Clerico et al., 2023), are typically executed using complex, automated laboratory platforms (Neumann et al., 2019). However, the urgent diagnostic needs in resource-limited settings underscore the necessity for high-sensitivity point-of-care testing (POCT). Lateral flow immunoassays (LFIA) are favored for their simplicity, affordability, and adherence to the WHO’s “ASSURED” criteria for diagnostics (Budd et al., 2023). Nonetheless, conventional LFIA utilizing gold nanoparticles for colorimetric detection often exhibit a higher LoD (>0.1 mg/L) (Liu et al., 2021; Awiaz et al., 2023; Truong et al., 2023), inadequate for hs-cTn detection, and their reliance on visual or standard commercial readers limits quantitative precision at the point of care. This scenario necessitates the development of advanced nano-platforms to enhance the sensitivity and specificity of LFIA for low concentration cTn detection, thereby improving the timeliness and accuracy of MI diagnosis (Rink and Baeumner, 2023).

Quantum dots (QDs) have been identified as superior alternatives to gold nanoparticles in LFIA systems due to their exceptional quantum yield, narrow emission spectra, and tunable optical properties (Lin et al., 2024; Gao et al., 2022). Encapsulating multiple QDs within a single carrier not only amplifies signal intensity but also reduces environmental interference and improves stability (Fang et al., 2022; Gao et al., 2024). Recent studies have delved into various encapsulation techniques, such as soft-template methods involving the self-assembly of amphiphilic polymers to envelop QDs (Huang et al., 2024), and hard-template methods where QDs are sequentially assembled on the surface of silica nanoparticles via a layer-by-layer approach (Li et al., 2025; Yang et al., 2023). Notably, dendritic mesoporous silica nanoparticles (DMSNs) offer a high surface area that significantly boosts QD loading capacity (Gao et al., 2021a; Zhu et al., 2024). However, the efficacy of LFIA is influenced by a multifaceted interaction of physical, chemical, and optical properties which impact signal intensity and analyte capture efficiency (Zhan et al., 2017; Bishop et al., 2019). Our previous work indicated that while larger DMSNs can accommodate more QDs, they might degrade assay performance by elevating background noise and diminishing capture efficiency through slower diffusion rates. Conversely, smaller DMSNs, while cost-effective and stable, have a limited QD loading capacity due to reduced surface area (Gao et al., 2021b). To address this, enhancing the pore size of these smaller DMSNs presents a viable solution. The application of sodium trifluoroacetate (FC_2_) as a structure-directing agent has shown promise in synthesizing small-sized, large-pore DMSNs for enzyme loading (Wang et al., 2016), yet its application in enhancing QD loading for LFIA is largely unexplored.

The integration of smartphones into LFIA systems has transformed the landscape of POCT by enhancing system portability and accessibility. Smartphones serve as multifunctional tools for control, analysis, and display, leveraging their high-resolution cameras and computational power to analyze test images, often rivaling benchtop readers (Xiao et al., 2022). Innovations include a smartphone-based system for Zika virus NS1 protein detection using a 3D-printed accessory for noise reduction and ImageJ for data analysis (Rong et al., 2019), and the use of an R Shiny application with a 3D-printed photo box for a more tailored analytical workflow (Schary et al., 2022). Moreover, Python-based tools have been developed for automated POCT quantification, emphasizing automation, reproducibility, and cost-effectiveness (Cuny et al., 2021). Despite these advancements, a critical gap remains in Python-based algorithms specifically designed for detecting faint signals on smartphone-adapted LFIA platforms, particularly when test strip signals are weak or when background autofluorescence and noise interferes with detection. This limitation constrains the minimum detectable concentration of analytes and underscores the urgent need for tools capable of intelligent image processing and adaptive detection of fluorescent test lines, thereby facilitating the sensitive detection of low-concentration analytes for early-stage diagnosis.

**SCHEME 1:**
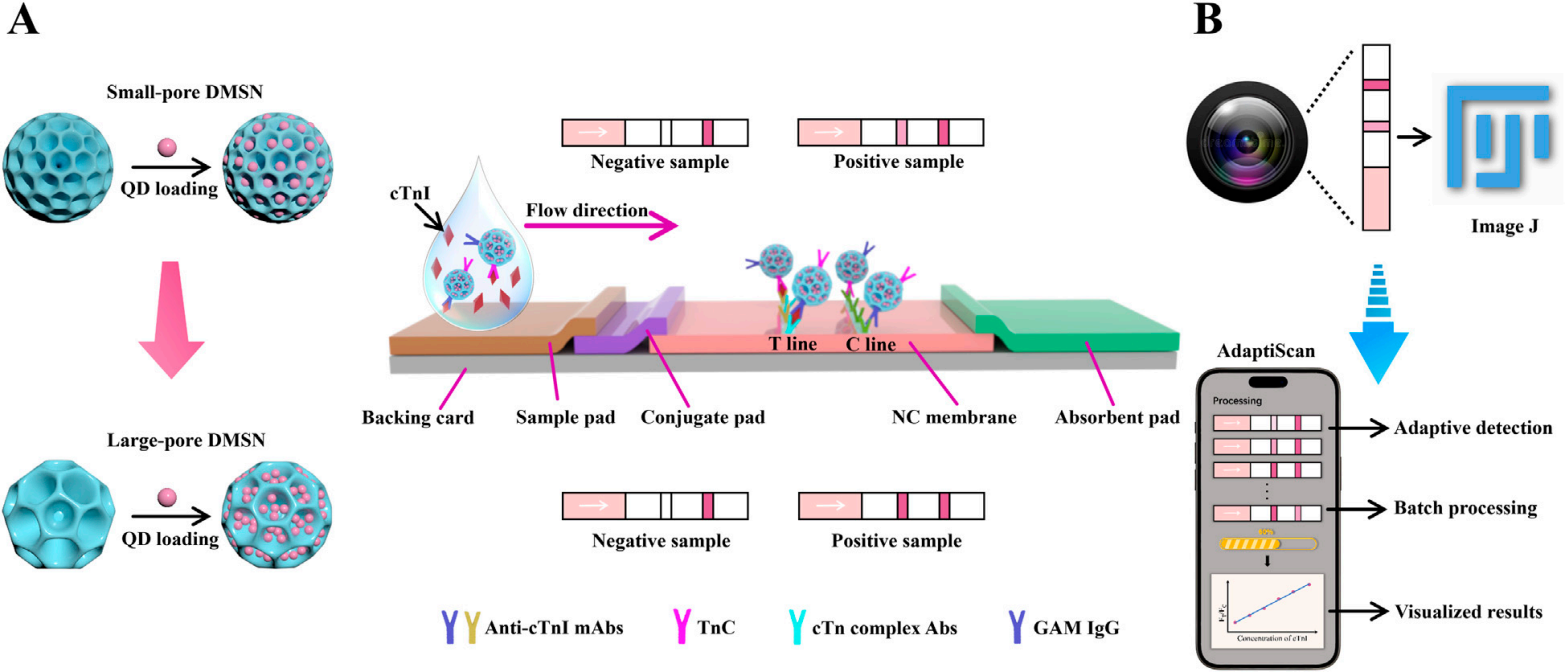
**(A)** Schematic illustration of the enhanced loading capacities of QDs in large-pore DMSN as compared to small-pore DMSN, along with the design of a DMSN-QD-based LFIA test strip for the detection of cTnI. **(B)** Schematic illustration of our custom-developed smartphone-based detection system equipped with adaptive detection algorithms for improved signal processing, named “AdaptiScan”, in comparison with the conventional ImageJ software.

In this study, we present an innovative LFIA platform for detecting cardiac troponin I (cTnI), utilizing small-sized, large-pore DMSNs (DMSN-2) with an average diameter of 122.3 nm and a pore size of 22.9 nm to significantly increase QD loading (Scheme 1A). This approach enhanced the QD loading capacity from 0.881 to 1.427 g QD/g silica compared to smaller pore DMSNs (DMSN-1, pore size 11.2 nm). We combined this nano-LFIA with a cost-effective miniaturized smartphone-based detection system named “AdaptiScan” (Scheme 1B). Using a Samsung Galaxy S9 in PRO mode (F1.5 aperture, ISO 640), we captured and processed fluorescence signals through a custom mobile application featuring adaptive detection algorithms, achieving an exceptional LoD of 42.6 ng/L for cTnI. This system also supports batch processing, thereby advancing the sensitivity and practicality of LFIA for cardiac biomarker detection in resource-constrained environments.

## 2 Materials and methods

### 2.1 Materials

All chemicals were used as received, without further purification. CdSe/ZnS QDs emitting at 620 nm were sourced from Xiamen Bohr Science & Technology Co., Ltd. Triethanolamine (TEA), cetyltrimethylammonium bromide (CTAB), (3-mercaptopropyl)trimethoxysilane (MPTMS), 3-mercaptopropionic acid (3-MPA), D-(+) sucrose, polyvinylpyrrolidone (PVP, Mw ≈ 24 K) and bovine serum albumin (BSA) were obtained from Adamas-beta. Phosphate-buffered saline (PBS) and FC_2_ were received from Sigma-Aldrich. Chloroform and hydrochloric acid (37% wt) were received from Sinopharm Chemical Reagent Co., Ltd. Poly(ethylene glycol) (PEG, Mw ≈ 1,500), tetraethyl orthosilicate (TEOS), sodium salicylate (NaSal), methanol, and ethanol were obtained from General-reagent, Tween 20 was purchased from TCI (Shanghai) Development Co., Ltd. Dimethyl sulfoxide (DMSO) was purchased from Shanghai Aladdin Bio-Chem Technology Co., Ltd. Ammonium hydroxide (28 wt.%) were received from Chem Supply Pty Ltd. The cardiac troponin I (cTnI) antigen (No. CTNI-Ag5) was obtained from FAPON Biotech Inc. Capture antibodies, including recombinant monoclonal anti-cTnI antibodies (anti-cTnI mAb, No. RC4T21-Y302) and human native cardiac troponin complex antibody (cTn complex Abs, No. 4TC2-20C6cc), along with detection antibodies such as recombinant anti-cTnI mAb (No. RC4T21-RecR85) and troponin C cardiac (TnC, No. 4T27cc-7B9cc), were obtained from HyTest. Human Immunoglobulin G (Human IgG) and goat anti-mouse (GAM) IgG were purchased from Changsha Bioadvantage Co. Ltd. Carcinoembryonic antigen (CEA), human alpha-fetoprotein (AFP), prostate-specific antigen (PSA), and C-reactive protein (CRP) antigens were obtained from Shanghai Linc-Bio Science Co., Ltd. Human serum was obtained from Jiangsu Kewei Biotechnology Co., Ltd. Materials for lateral flow assays included nitrocellulose (NC) membrane (CN140), absorbent pad, sample pad, conjugate pad and PVC backing card, all of which were purchased from Shanghai Kinbio Tech Co., Ltd. Materials for the smartphone-based detection device include UV lamps, which were sourced from Shenzhen Orange Purple Lighting Optoelectronics Co., Ltd., and filter glasses and prisms, which were purchased from Shenzhen Hengyang Optical Co., Ltd. Deionized water (DI water) used for all experiments was prepared using a Milli-Q system (Millipore, Bedford, MA).

### 2.2 Synthesis of nanomaterials

#### 2.2.1 Synthesis of SNPs

In a typical synthesis, uniform nonporous Stöber spheres (SNPs) were synthesized according to a previously reported method (Yu et al., 2016). Absolute ethanol (17.6 mL) was mixed with Milli-Q water (6.8 mL) and ammonium hydroxide (28 wt.%, 0.7 mL) at 25°C. Subsequently, a mixture of TEOS (2.8 mL) and ethanol (22.2 mL) was added under vigorous magnetic stirring. After 13 h, the synthesized SNPs were separated by centrifugation and washed with ethanol. The SNPs were finally dispersed in ethanol for further use.

#### 2.2.2 Synthesis of DMSN-1

Monodispersed DMSN-1 were synthesized with modifications to a reported method (Gao et al., 2021b). TEA (408 mg) was added to 150 mL of water and stirred at 80°C for 30 min. Afterwards, 2.28 g of CTAB and 0.403 g of NaSal were introduced and stirred for another hour. 24 mL of TEOS and 0.76 mL of ethanol were then added, maintaining stirring for an additional 2 h. The DMSN-1 were collected via centrifugation and washed with ethanol to remove residual reactants. Subsequently, the surfactant was extracted using acidic methanol (3 mL of 37% HCl in 50 mL of absolute methanol) at 60°C for three 6-h sessions. The resulting DMSN-1 were dispersed in ethanol.

#### 2.2.3 Synthesis of DMSN-2

DMSN-2 were synthesized similarly to DMSN-1 but with a different porogen (Wang et al., 2016; Wang et al., 2018). TEA (408 mg) was dissolved in 150 mL of water and stirred at 80°C for 30 min followed by the addition of CTAB (2.28 g) and FC_2_ (0.8509 g), stirring for 1 h. After the addition of 24 mL of TEOS, the solution was further stirred for 1 h before product was collected. Subsequently, the surfactant was extracted with acidic methanol as described for DMSN-1. DMSN-2 were then dispersed in ethanol.

#### 2.2.4 Thiol modification of nanomaterials

For surface modification with thiol groups, 40 mL of ethanol solution containing 400 mg of nanomaterials was mixed with 1 mL of ammonia and 480 μL of MPTMS. This mixture was stirred at room temperature for 12 h. The modified nanoparticles were collected by centrifugation at 10,000 rpm for 20 min and washed three times with ethanol. The obtained thiolated DMSN (T-DMSN) were dispersed in 40 mL of ethanol.

#### 2.2.5 Preparation of fluorescent nanomaterials

To immobilize QDs in T-DMSN, a volume of CdSe/ZnS QDs in chloroform and T-DMSN in ethanol were sonicated for 10 min (Gao et al., 2021b). The resulting DMSN-QD composites were centrifuged and washed multiple times with chloroform to remove excess QDs. The precipitate was redispersed in 3.5 mL of chloroform, mixed with an equal volume of DMSO and 25 μL of 3-MPA, and sonicated for 30 min to facilitate phase transfer (Gao et al., 2021b). The carboxyl-terminated DMSN-QD-COOH were then collected, washed with DI water, and dispersed in DI water for further use.

#### 2.2.6 Preparation of immuno-fluorescent nanomaterials

DMSN-QD-COOH were functionalized with anti-cTnI mAbs and TnC through physical adsorption (Gao et al., 2021b). A quantity of DMSN-QD-COOH was suspended in 1 mL of PBS buffer (10 mM, pH = 7.4), followed by the addition of 10 μl of anti-cTnI mAbs and TnC (1 mg/mL each). After 2 h of gentle agitation at room temperature, the immuno-fluorescent DMSN-QD-COOH (I-DMSN-QD-COOH) were harvested by centrifugation, washed once with PBS containing 0.05% Tween-20 (PBST), and blocked using a buffer containing 10 mM PBS, 10% BSA, and 0.5% Tween-20 for 2 h. The final preparation was redispersed in 20 mM PBS buffer with 1% sucrose, 2.5% BSA and 2% PEG-1500 and stored at 4°C.

### 2.3 Development of LFIA method for cTnI detection

#### 2.3.1 Fabrication of LFIA

The sample pads and conjugation pads were treated with 20 mM phosphate buffer (pH = 7.4) containing 1% BSA (w/v), 1% Tween 20 (v/v), 2.5% sucrose and 0.3% PVP K24 (w/v) for 1 min and dried at 37°C. For the test line (T line), a mixture of cTn complex Abs and anti-cTnI mAbs (0.5 mg/mL each) was dispensed onto the NC membrane at 1.0 μL/cm. The C line was prepared with goat anti-mouse IgG (GAM IgG, 1 mg/mL) at the same rate. The NC membrane was then dried at 37°C for 2 h. The pads and NC membrane were assembled onto a PVC backing card and cut into 4 mm wide strips.

#### 2.3.2 Fabrication of smartphone-based test strip detection device

The smartphone-based detection device consists of a 3D-printed holder that integrates several critical optical components: an ultraviolet light-emitting diode (UV LED) with a wavelength of 365–370 nm, an aluminum UV reflective mirror, a 600 nm long-pass filter, and a Samsung Galaxy S9 smartphone (Figure 4A). The holder is designed with a base slot for the exact placement of the test strip. The long-pass filter serves to attenuate interference from both the UV excitation light and background autofluorescence. The UV LED sits on an adjustable base to achieve optimal collimation of light. For image acquisition, the Samsung Galaxy S9’s camera utilizes manual photographic controls, with images taken from the top of the holder. A 25 mm × 25 mm reflective mirror is employed to provide even illumination by redirecting horizontally scattered excitation light onto the test strips positioned at the bottom of the holder.

#### 2.3.3 Detection of cTnI using immuno-fluorescent nanomaterial-based LFIA

A mixture of 97 µL cTnI standard solution and 3 µL I-DMSN-QD-COOH was applied to the sample pad of the LFlA, allowing all liquid to migrate to the absorbent pad by capillary action. After 15 min, the assay was evaluated using the smartphone-based test strip detection device. All groups performed in triplicate.

### 2.4 Materials characterization

Transmission electron microscopy (TEM), high-resolution TEM (HRTEM) images and energy-dispersive X-ray spectroscopy (EDS) elemental mappings were acquired with a Thermo Talos F200X instrument. For TEM analysis, samples were prepared by dispersing the powders in ethanol with sonication, followed by deposition and drying on the holey carbon film supported by copper grids. The morphology of the samples was observed with a Hitachi SU8010 field-emission scanning electron microscopy (FE-SEM) operated at 5 kV. SEM specimens were prepared by dispersing the powders in ethanol, then depositing them onto aluminum foil pieces, which were subsequently attached to a conductive carbon film on the SEM mount. The SEM mount was dried in a vacuum oven at 60°C for 12 h prior to characterization. The specific surface area and pore size distribution were determined using the Brunauer–Emmett–Teller (BET) method and the Barrett–Joyner–Halenda (BJH) method derived from the adsorption branch of the nitrogen isotherms, respectively. The loading amount of QDs was evaluated by measuring the concentration of cadmium (Cd) atoms in the synthesized materials using Thermo Fisher Scientific iCAP RQ inductively coupled plasma mass spectrometry (ICP-MS). Zeta potential and dynamic light scattering (DLS) measurements were carried out in DI water at 25°C using a Malvern Zetasizer Nano ZEN3700. Fluorescence emission spectra were record on a Hitachi F-2710 spectrofluorometer, with scanning from 550 to 700 nm under a fixed excitation wavelength of 400 nm. UV-vis absorption spectra were acquired with a Shimadzu UV-3600i plus over the range of 550–700 nm at room temperature.

## 3 Results and discussion

### 3.1 Characterization of silica nanoparticles

**FIGURE 1:**
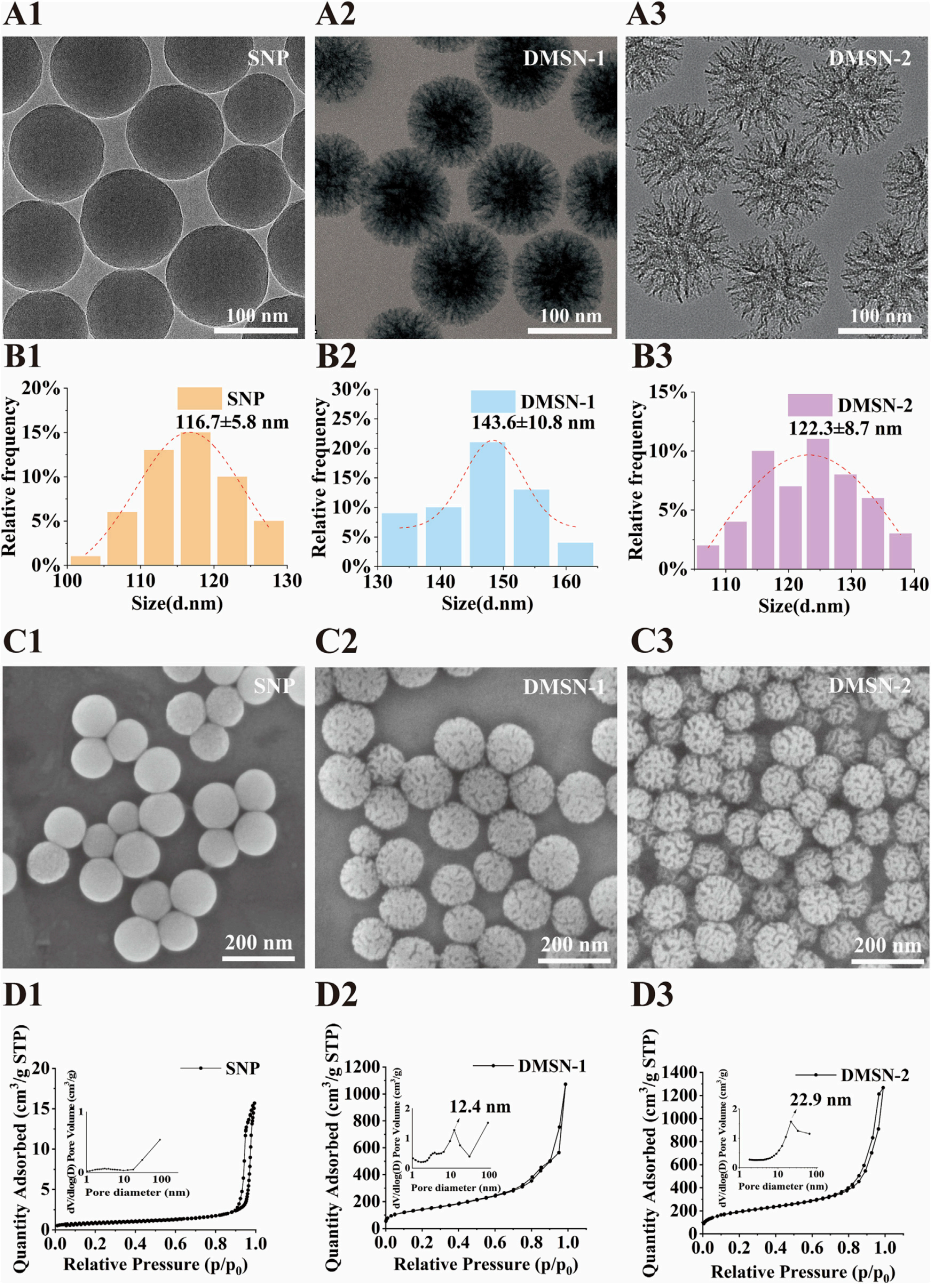
TEM images of **(A1)** SNP, **(A2)** DMSN-1 and **(A3)** DMSN-2. Corresponding TEM size distributions and Gaussian fittings for **(B1)** SNP, **(B2)** DMSN-1 and **(B3)** DMSN-2. SEM images of **(C1)** SNP, **(C2)** DMSN-1 and **(C3)** DMSN-2. N_2_ sorption isotherms and pore size distribution curves for **(D1)** SNP, **(D2)** DMSN-1 and **(D3)** DMSN-2.

To investigate the effect of pore size on QD loading, three types of silica nanoparticles were synthesized: non-porous silica nanoparticles (SNP), small pore-sized mesoporous silica nanoparticles (DMSN-1), and large pore-sized mesoporous silica nanoparticles (DMSN-2). TEM images (Figures 1A1–A3) demonstrate that all nanoparticles are monodisperse with similar overall sizes but distinct pore structures. The average particle sizes from TEM analysis are 116.7 ± 5.8 nm for SNP, 143.6 ± 10.8 nm for DMSN-1, and 122.3 ± 8.7 nm for DMNS-2 (Figures 1B1–B3). The SNPs were prepared using the Stöber method (Yu et al., 2016), known for producing homogenous, non-porous silica spheres. DMSN-1 was synthesized with CTAB and NaSal as structure-directing agents. The salicylate anions (Sal^−^) from NaSal facilitate pore formation by inducing micelle swelling, resulting in pores with an average size of approximately 12.4 nm (Yang et al., 2016). In contrast, the synthesis of DMSN-2 employed CTAB alongside a fluorocarbon surfactant (FC_2_) as structure-directing agents (Wang et al., 2016), leading to an increase in pore size to 22.9 nm due to enhanced micelle interactions. The fluorocarbon anions interact with the positively charged head groups of CTA^+^ in CTAB, penetrating the hydrophobic core of the micelles. This interaction increases the packing parameter (g), leading to a structural transition from swollen micelles to lamellar structures, thus promoting the formation of larger pores. Comparatively, NaSal, with its phenolic group and lower hydrophobicity, forms smaller pores due to a more compact micelle structure. SEM images (Figures 1C1–C3) support these findings, showing a transition from a smooth surface in SNP to increasingly porous surfaces in DMSN-1 and DMSN-2. BET analysis (Figures 1D1–D3; Supplementary Table 1) reveals an increase in specific surface areas from 380.9 m^2^/g for SNP (no pores) to 549.8 m^2^/g for DMSN-1 (12.4 nm pores) and 666.3 m^2^/g for DMSN-2 (22.9 nm pores), suggesting a direct correlation between pore size and surface area, which can enhance QD loading capabilities. DLS measurements reveal hydrodynamic diameters of 144.9 ± 2.1 nm, 170.5 ± 2.5 nm, and 185.0 ± 8.6 nm, with polydispersity indices (PDI) of 0.031 ± 0.019, 0.040 ± 0.013, and 0.262 ± 0.009 for SNP, DMSN-1, and DMSN-2, respectively. The zeta potential values are measured to be −25.6 ± 1.3 mV, −25.0 ± 0.9 mV, and −22.6 ± 1.3 mV (Supplementary Table 1).

### 3.2 QD loading capacity

**FIGURE 2:**
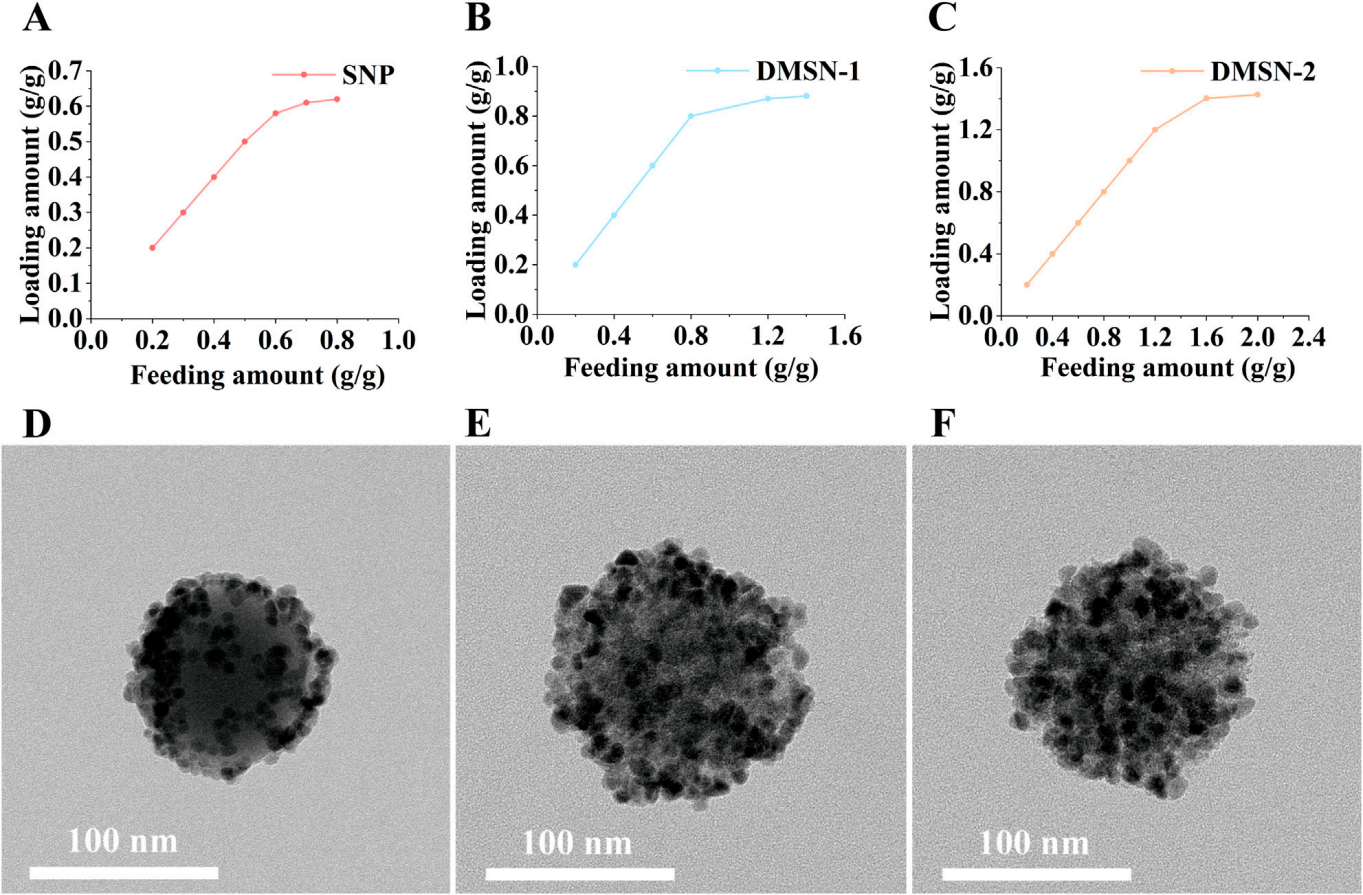
Relationships between QD loading and QD feeding amount for **(A)** SNP, **(B)** DMSN-1, and **(C)** DMSN-2, with corresponding TEM images for **(D)** SNPQD, **(E)** DMSN-1-QD, and **(F)** DMSN-2-QD.

The QD loading capacity was assessed for silica nanoparticles with varying pore sizes. The QDs, which emit at 620 nm (Supplementary Figure 1A) and have a TEM size distribution of 12.5 ± 2.4 nm (Supplementary Figure 1B), were immobilized via thiol-metal coordination. As illustrated in Figures 2A–C, QD loading increased with feeding amount across all groups until reaching saturation. Quantitative analysis by ICP-MS reveals maximum QD loading capacities of 0.620, 0.881, and 1.427 g QD/g silica for SNP, DMSN-1, and DMSN-2, respectively. This enhancement in loading capacity aligns with the increase in the surface area provided by larger pores, consistent with previous studies (Zhu et al., 2024; Gao et al., 2021b; Huang et al., 2018). Specifically, DMSN-2 exhibits the highest capacity due to its larger pore size. As can be seen from Figure 2D, the QDs are on the surface of the non-porous SNP. With an increase in pore size to 12.4 nm for DMSN-1, more QD can be immobilized (Figure 2E). Pore blocking in DMSN-1 is evidenced by the SEM image presented in Supplementary Figure 2A due to similar QD and pore size. An increase in pore size to 22.9 nm allows DMSN-2 to maintain open pores for further QD loading (Supplementary Figure 2B; Figure 2F). Given its lower QD loading capacity, SNP was excluded from further studies.

### 3.3 Characterization of DMSN-QD-COOH

**FIGURE 3:**
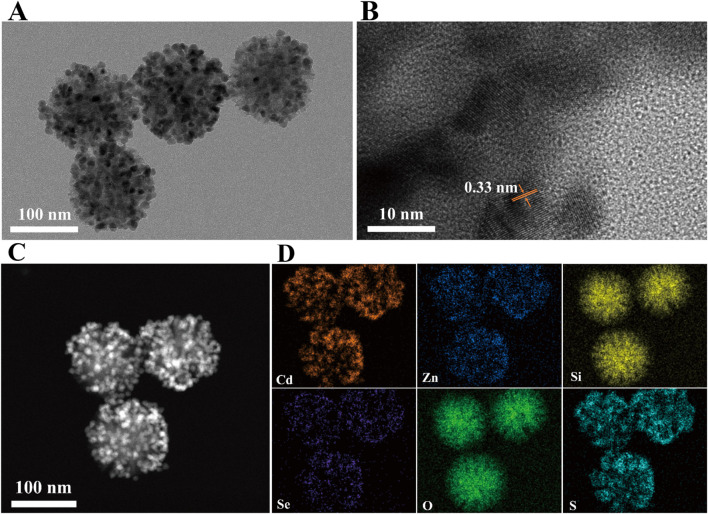
**(A)** TEM image, **(B)** HRTEM image, **(C)** HAADF-STEM image, and **(D)** EDS elemental mapping of DMSN-2-QD-COOH.

To ensure the suitability of the prepared DMSN-QD nanocomposites for LFIA application, a 3-MPA ligand exchange strategy was implemented to transfer DMSN-QD into an aqueous phase (Gao et al., 2021b). The substitution of hydrophobic trioctylphosphine oxide and oleic acid (TOPO/OA) ligands on the QD surface with 3-MPA can introduce surface defects and lead to fluorescence quenching (Gao et al., 2021a). Therefore, the amount of 3-MPA was carefully optimized to minimize fluorescence quenching while ensuring effective phase transfer (Supplementary Figure 3). The optimal volumes of 3-MPA for DMSN-1-QD and DMSN-2-QD are determined to be 27.5 µL and 30.0 µL, respectively. The resulting DMSN-2-QD-COOH displays both high fluorescence and excellent water dispersibility. TEM and HRTEM images (Figures 3A, B) depict a pomegranate-like structure with crystalline QDs embedded within the silica matrix. The lattice spacing of 3.3 Å corresponds to the (100) plane of wurtzite ZnS (Xia et al., 2010). The high-angle annular dark-field scanning transmission electron microscopy (HAADF-STEM) image (Figure 3C) shows numerous bright spots, indicative of high loading and homogeneous distribution of CdSe/ZnS QDs within the silica matrix. This is further confirmed by the EDS elemental mappings showing Cd, Zn, Si, Se, S, and O (Figure 3D).

### 3.4 Analytical performance of I-DMSN-QD-COOH

**FIGURE 4:**
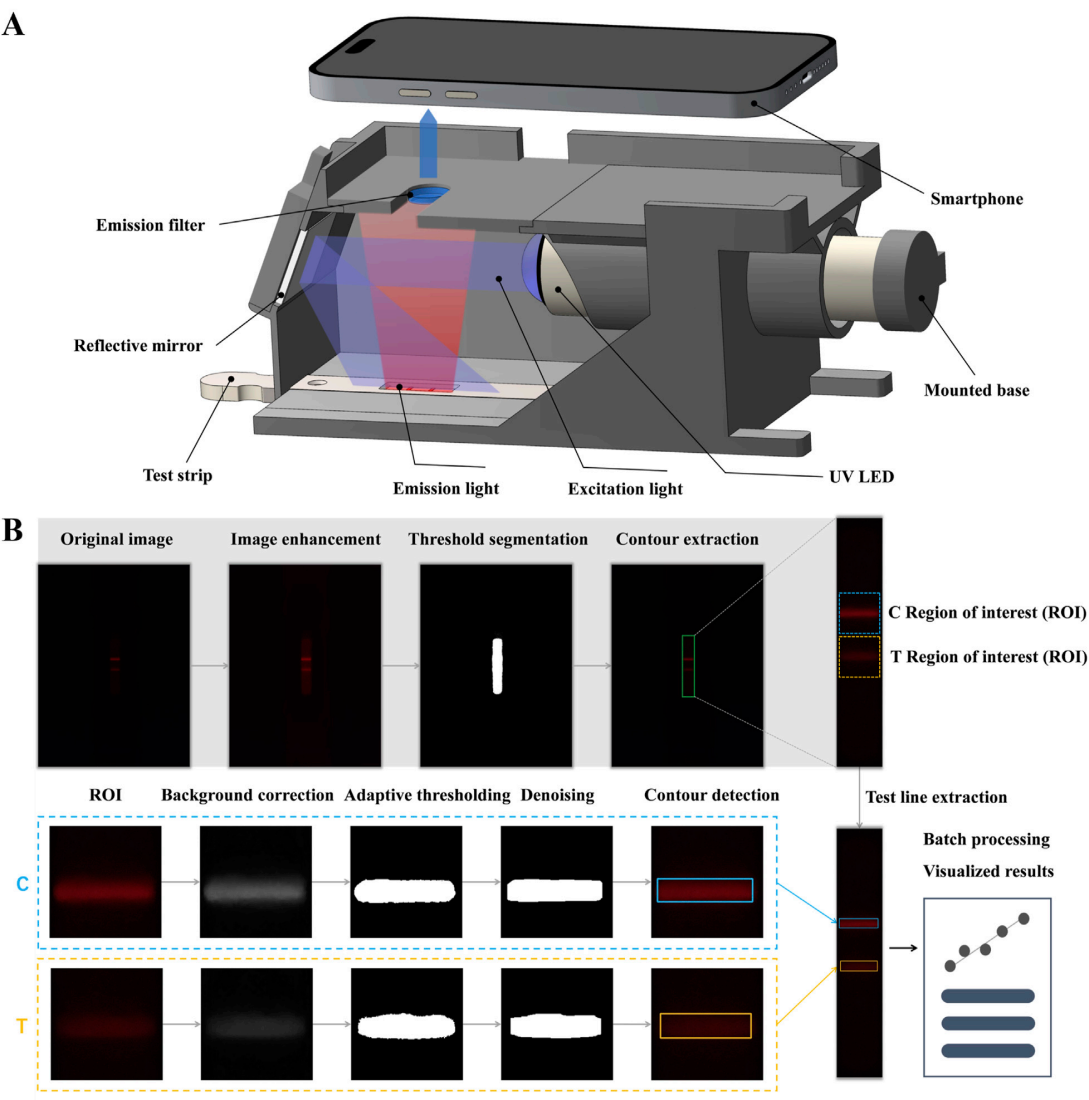
Schematic illustration of “AdaptiScan” system. **(A)** Demonstration of the smartphone-based optical device for test strip detection. **(B)** Flowchart of the adaptive detection algorithms for fluorescent signal extraction and image processing of the reacted test strips.

The high fluorescence intensity and excellent aqueous dispersibility of DMSN-QD-COOH render them suitable for LFIA applications. By conjugating DMSN-QD-COOH with cTnI detection antibodies, I-DMSN-QD-COOH were synthesized for the detection of cTnI, a critical biomarker for MI. The assay procedure involves applying 100 µL of a premixed cTnI standard solution with I-DMSN-QD-COOH to the sample pad, resulting in specific antigen-antibody interactions at the T line, forming a fluorescent sandwich structure. C line validation is ensured through interactions with GAM IgG. The quantification of fluorescence signals at the T and C lines is facilitated by our “AdaptiScan” system, employing a Samsung Galaxy S9 smartphone for image capture and analysis in PRO mode, configured with an F1.5 aperture and ISO 640 settings. Fluorescence signals from the reacted test strips are recorded as RGB images by the smartphone’s camera above a long-pass filter (Figure 4A). The captured images are then processed and analyzed via a custom-developed mobile application featuring adaptive detection algorithms for precise evaluation of the fluorescent intensities at both the T and C lines (Figure 4B).

The initial processing step involves image enhancement, threshold segmentation, and contour extraction to accurately locate the test strip and define the regions of interest (ROI) for T and C lines adaptively (Figure 4B). Specifically, the contrast-limited adaptive histogram equalization (CLAHE) algorithm is applied to enhance the contrast of the red channel in the RGB images, thereby improving visibility of the test strip region. For quantitative assessment of T and C line fluorescence, a morphological opening algorithm is utlized to estimate background noise, which is then subtracted from the red channel for background correction. Adaptive thresholding using Otsu’s method further refines the identification of T and C line regions within the ROI. Subsequent steps include smoothing and morphological denoising to accurately delineate the T and C line contours while reducing background noise interference. The average grayscale value of pixels within these delineated areas is then calculated to quantify fluorescence. The above regulated procedure significantly enhances the detection of weak fluorescent signals against a complex and noisy background on FLIA test strips, demonstrating excellent robustness and stability for comprehensive POCT applications. Our application is also adept at automatically processing images from different batches and samples, thereby enhancing its practical utility.

**FIGURE 5:**
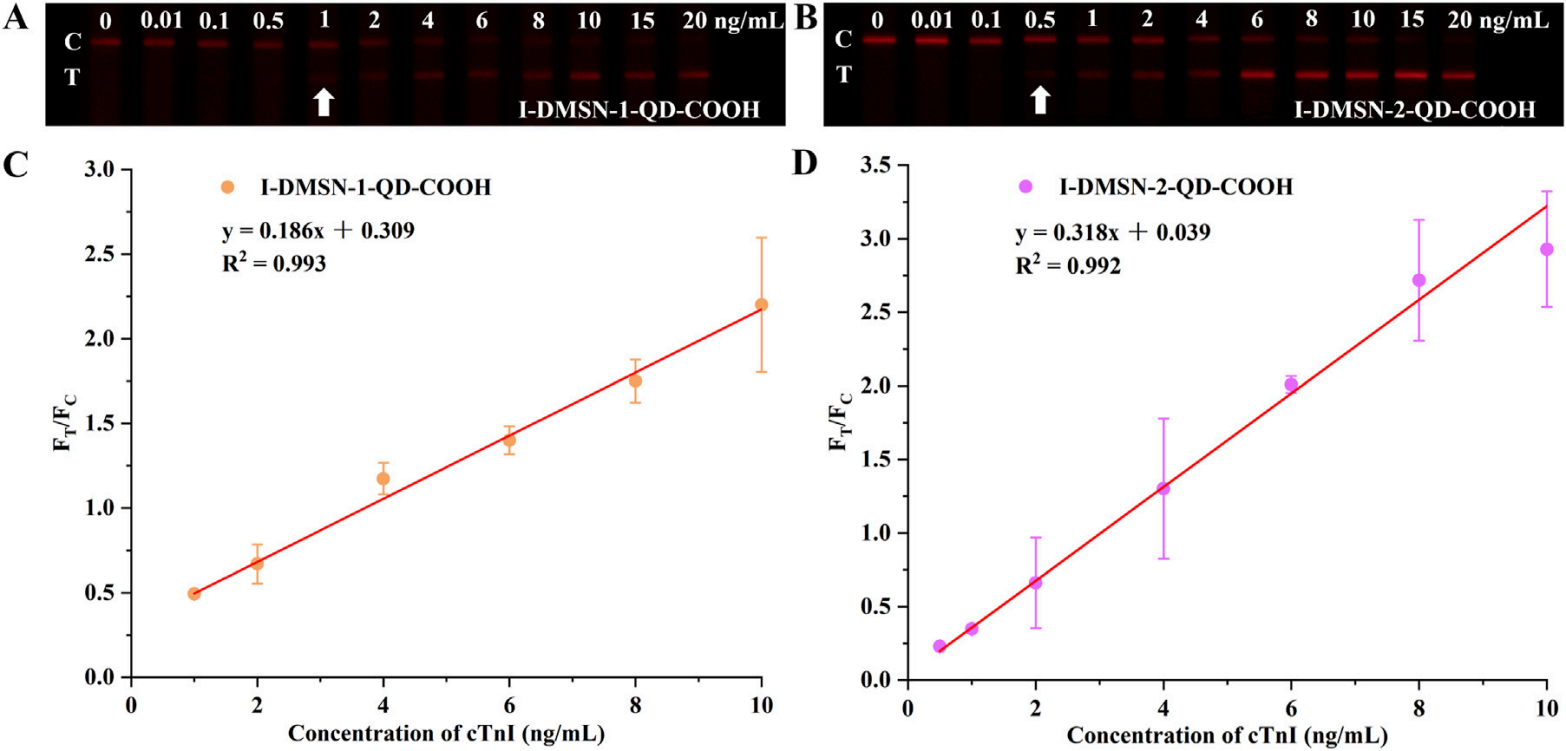
I-DMSN-QD-COOH-based LFIA for sensitive cTnI detection. Fluorescence images of test strips for **(A)** I-DMSN-1-QD-COOH and **(B)** I-DMSN-2-QD-COOH in the presence of varying cTnI concentrations from 0 to 20 ng/mL. Linear response curves for **(C)** I-DMSN-1-QD-COOH and **(D)** I-DMSN-2-QD-COOH-based LFIA with cTnI concentration ranges of **(C)** 1–10 ng/mL and **(D)** 0.5–10 ng/mL, respectively. Error bars represent the standard errors from three independent experiments.

The pore size of nanoparticles significantly influences QD loading capacity, which in turn affects the sensitivity of LFIA. To enhance detection sensitivity, the analytical performances of I-DMSN-QD-COOH with varying pore sizes were compared. Initially, the optimal concentration of the cTnI detection antibody for conjugation was determined. The optimal detection antibody concentrations for forming I-DMSN-QD-COOH are found to be 30 μg/mL for I-DMSN-1-QD-COOH and 10 μg/mL for I-DMSN-2-QD-COOH (Supplementary Figure 4). The detection capabilities of these I-DMSN-QD-COOH-labeled LFIA were then evaluated across a cTnI concentration range of 0–20 ng/mL. As illustrated in Figures 5A, B, all LFIA strips yield valid results with visible red fluorescent C lines. The T line intensity increases with cTnI concentrations from 0 to 20 ng/mL (diluted in PBS). I-DMSN-2-QD-COOH displays a lower LoD of 500 pg/mL, compared to 1 ng/mL for I-DMSN-1-QD-COOH as determined by naked eye observation. The F_T_/F_C_ value is utilized for cTnI quantification, with Figures 5C, D displaying a linear relationship with concentration from 1 to 10 ng/mL for I-DMSN-1-QD-COOH and from 0.5 to 10 ng/mL for I-DMSN-2-QD-COOH. According to the 3σ/S rule (where σ is the standard deviation of the blank and S is the slope of the linearity range), the LoDs for I-DMSN-2-QD-COOH and I-DMSN-1-QD-COOH are calculated to be 42.6 ng/L and 436.7 ng/L, respectively. The combination of a low LoD (42.6 ng/L) and a wide linear detection range (0.5–10 ng/mL) make I-DMSN-2-QD-COOH the superior label for LFIA-based cTnI detection. Notably, the current assay demonstrates higher sensitivity compared with reported LFIA strips for cTnI analysis (Supplementary Table 2)

### 3.5 Robustness of the I-DMSN-2-QD-COOH -labelled LFIA for clinical application

**FIGURE 6:**
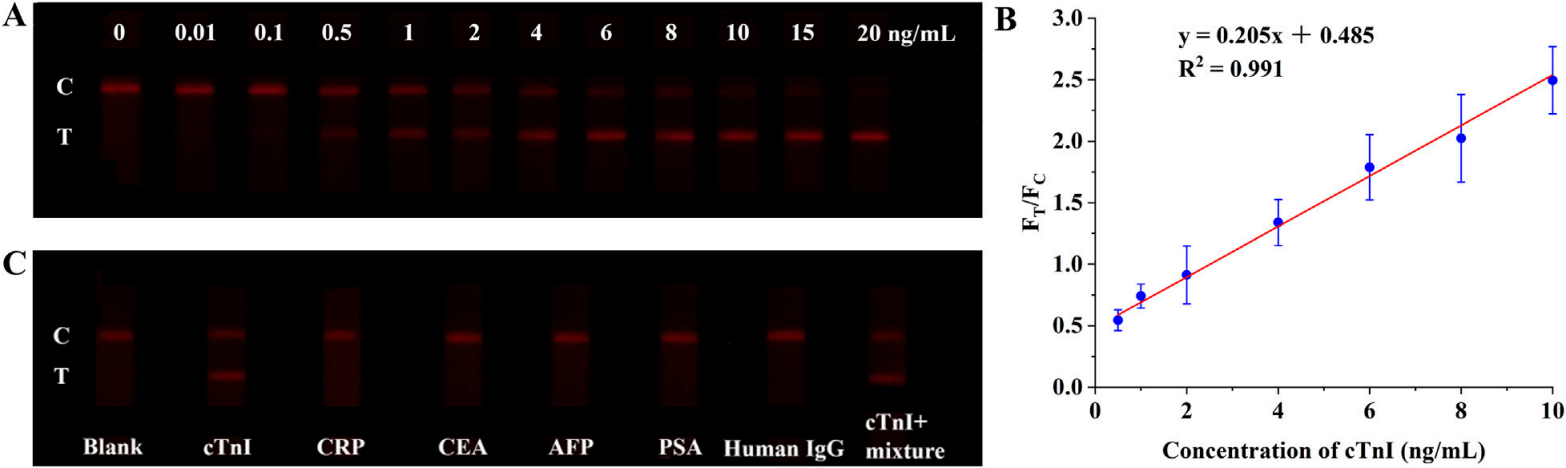
**(A)** Fluorescence photographs of test strips using I-DMSN-2-QD-COOH in human serum with cTnI concentrations from 0 to 20 ng/mL. **(B)** Linear response curve of the I-DMSN-2-QD-COOH-based LFIA for cTnI detection in human serum, spanning concentrations from 0.5 to 10 ng/mL. Error bars denote the standard errors from three independent experiments. **(C)** Fluorescence photographs evaluating the specificity of the I-DMSN-2-QD-COOH-labelled LFIA with various protein analytes at 10 μg/mL.

The practical utility of the I-DMSN-2-QD-labeled LFIA for detecting cTnI in clinical settings was assessed using human serum samples. Consistent with the results in PBS buffer (Figure 5B), the LFIA strips labeled with I-DMSN-2-QD-COOH produce valid results with visible C and the T line intensity increased in a concentration-dependent manner with cTnI concentrations ranging from 0 to 20 ng/mL, as depicted in Figure 6A. A linear correlation between the signal ratio and cTnI concentration is observed within the range of 0.5–10 ng/mL, with a coefficient of determination (R^2^) of 0.991 (Figure 6B). While our assay’s LoD of 42.6 ng/L slightly exceeds the 99th percentile URL of 40 ng/L (Mariathas et al., 2019), this LoD remains suitable for identifying clinically significant elevations in acute settings. Further optimization is required to achieve the sensitivity needed for early MI detection. To evaluate the specificity of the I-DMSN-2-QD-COOH-labeled LFIA, interference tests were conducted with various proteins including CRP, CEA, AFP, PSA, and human IgG. As shown in Figure 6C, none of these interfering proteins produces a detectable signal even at concentrations 10,000 times higher than cTnI. In contrast, a cTnI concentration of 1 ng/mL resulted in a strong fluorescent signal despite the presence of high levels of mixed interfering proteins. This highlights the high specificity of the current assay for cTnI, indicating its potential for accurate clinical diagnosis of MI. Considering that low cost is one of the main strengths of LFIA, we have included a comparison with commercial products for cTnI in Supplementary Table 3. The cost of the DMSN-2-QD-COOH-labelled LFIA was estimated (see Supplementary Table 4). The low cost of ¥2.27/strip indicates the feasibility for potential commercial applications.

## 4 Conclusion

In this study, three types of silica nanoparticles with varied pore sizes were synthesized to optimize QD loading for enhancing the sensitivity of LFIA. The small-sized and large-pore mesoporous silica nanoparticles (DMSN-2) demonstrated the highest QD loading capacity of 1.427 g QD/g silica. Integration with our custom “AdaptiScan” smartphone-based detection system, which employs adaptive detection algorithms for image processing and fluorescent signal extraction, a low LoD of 42.6 ng/L for cTnI was achieved, meeting the criteria for clinical diagnostic applications. Our findings provide valuable insights into the rational design of high-quality, water-dispersible QD-based nanoplatforms and sensitive smartphone-based POCT devices, thereby facilitating the development of an ultrasensitive LFIA platform.

## Data Availability

The original contributions presented in the study are included in the article/Supplementary Material, further inquiries can be directed to the corresponding authors.
