# Supplementary material for: Enhancing lateral flow immunoassay performance for cardiac troponin I detection with pore-size tailored silica nanoparticles and smartphone-based “AdaptiScan” analysis
Source: Front Bioeng Biotechnol. 2025 Mar 26;13:1568719. doi: 10.3389/fbioe.2025.1568719 (PMC11978842; doi:10.3389/fbioe.2025.1568719)
Supplement: Supplementary file 1 [file Table1.doc]

Supplementary Material

# Enhancing Lateral Flow Immunoassay Performance for Cardiac Troponin I Detection with Pore-Size Tailored Silica Nanoparticles and Smartphone-Based "AdaptiScan" Analysis

Shaonian Ye^1^, Cifu Xu^2^, Huilin Li^3^, Shilun Feng^4^, Yan Wang^2*^, Fang Gao^1*^

^1^Institute of Energy Materials Science, University of Shanghai for Science and Technology, Shanghai 200093, China

^2^College of Information and Electrical Engineering, China Agricultural University, Beijing 100083, China

^3^Department of Nephrology, Gongli Hospital of Shanghai Pudong New Area, Shanghai 200135, China

^4^State Key Laboratory of Transducer Technology, Shanghai Institute of Microsystem and Information Technology, Chinese Academy of Sciences, Shanghai, 200050, China

*** Correspondence:**Yan Wang; Fang Gao
[yanwang@cau.edu.cn](mailto:yanwang@cau.edu.cn); fgao@usst.edu.cn

Supplementary Table 1. Specific Brunauer-Emmett-Teller (BET) surface areas, dynamic light scattering (DLS) measurements, polydispersity indices (PDI), and Zeta potential values for SNP, DMSN-1 and DMSN-2.

| Sample | S_BET_ (m^2^ g^-1^) | Size (nm) | PDI | Zeta potential (mV) |
| --- | --- | --- | --- | --- |
| SNPs | 380.9 | 144.9±2.1 | 0.031±0.019 | -25.6±1.3 |
| DMSN-1 | 549.8 | 170.5±2.5 | 0.040±0.013 | -25.0±0.9 |
| DMSN-2 | 666.3 | 185.0±8.6 | 0.262±0.009 | -22.6±1.3 |


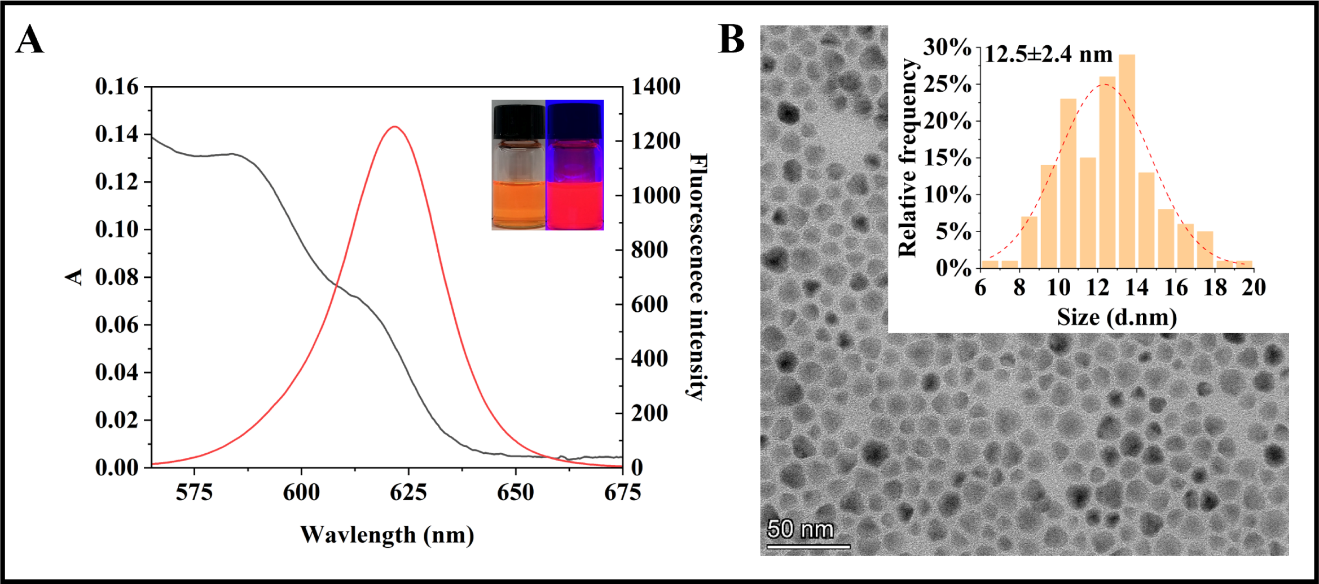


Supplementary Figure 1. (A) Fluorescence spectrum and UV-visible absorption spectrum of QDs. Insets show images of QDs under visible (left) and UV light (right). (B) TEM image of QDs with an inset for the corresponding TEM size distribution and Gaussian fitting.


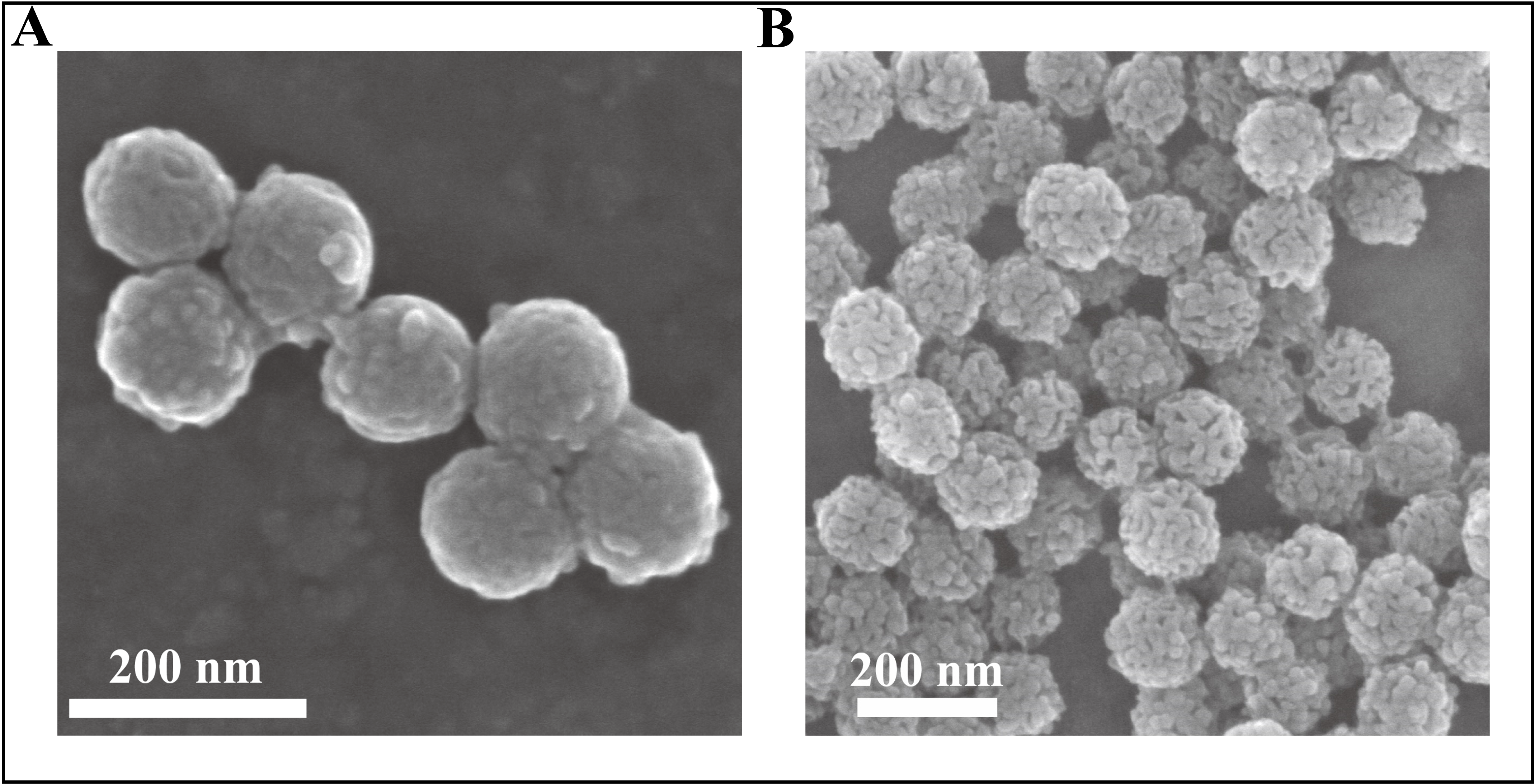


Supplementary Figure 2. SEM images of (A) DMSN-1-QD and (B) DMSN-2-QD.


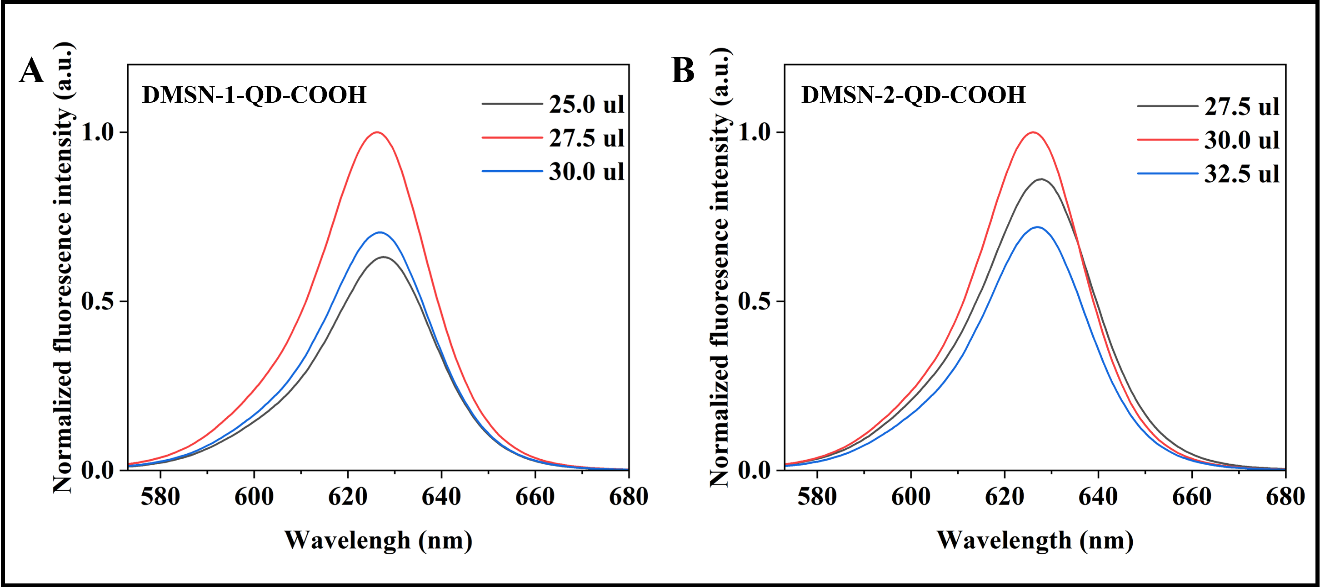


Supplementary Figure 3. Normalized fluorescence spectra of (A) DMSN-1-QD-COOH, and (B) DMSN-2-QD-COOH synthesized at different 3-MPA quantities.


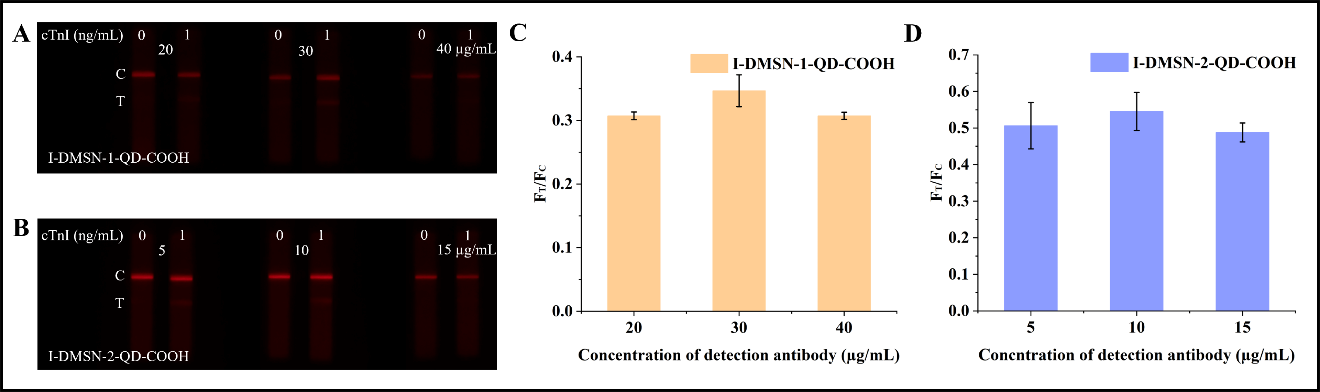


**Supplementary Figure 4.** Optimization of detection antibody concentrations. Fluorescence photographs of test strips are shown for (A) I-DMSN-1-QD-COOH and (B) I-DMSN-2-QD-COOH at cTnI concentrations of 0 and 1 ng/mL, utilizing different concentrations of cTnI detection antibody in the conjugation process. The ratios of fluorescence intensity between the test (T) line and control (C) line are presented for (C) I-DMSN-1-QD-COOH and (D) I-DMSN-2-QD-COOH.

Supplementary Table 2. Summary of the analytical performances of cTnI detection with LFIA strips.

| Labels | LoD^a^ | References |
| --- | --- | --- |
| Ctrate-capped Au@Ag-Au NPs | 90 | (Bai et al., 2018) |
| Nonspherical gap-enhanced Raman label | 100 | (Khlebtsov et al., 2019) |
| AuNP-polyHRP | 84 | (Han et al., 2020a) |
| Gold-ion amplification | 92 | (Han et al., 2020b) |
| Time-resolved fluorescence resonance energy transfer between raspberry-type europium particles and gold nanorod | 97 | (Lee et al., 2020) |
| DMSN-AuNP | 70 | (Li et al., 2025) |
| **I-DMSN-2-QD-COOH** | **42.6** | **This work** |

^a^Limit of detection (ng/L).

Supplementary Table 3. Price comparison of our method with commercial kits for cTnI

| Methods | Label | Price | LoD^a^ | Vendors |
| --- | --- | --- | --- | --- |
| LFIA | Gold NPs | 25.9 yuan/strip | 400 | Bioscience Diagnostic Technology Co., Ltd. |
| LFIA | Gold NPs | 16.0 yuan/strip | 500 | InTec Produce, Inc. |
| LFIA | Gold NPs | 19.9 yuan/strip | 500 | Hangzhou Alltest Biotech Co., Ltd. |
| LFIA | Gold NPs | 34.0 yuan/strip | 500 | Hangzhou Biotest biotech Co., Ltd. |
| LFIA | Gold NPs | 33.0 yuan/strip | 500 | Beijing Kewei clinical diagnostic reagent Inc. |
| ELISA | / | 650 yuan/48Test | 60 | Ji-ichi Biology Technology Co., Ltd. |
| ELISA | / | 850 yuan/48Test | 18 | Shanghai Chutai biology science and technology Co., Ltd |
| ELISA | / | 1200 yuan/48Test | 100 | Shanghai Xuanzekang Biology Co., Ltd |
| LFIA | DMSN-2-QD-COOH | 2.27 yuan/strip | 42.6 | Our work |

Supplementary Table 4. Estimated cost of the DMSN-2-QD-COOH labelled LFIA strip.

| Chemical/material name | CAS/product name | Size | Price (CNY) | Price/strip (CNY) |
| --- | --- | --- | --- | --- |
| TEA | 102-71-6 | 500 mL | 54 | 7.10×10^-8^ |
| CTAB | 57-09-0 | 100 g | 34 | 1.07×10^-6^ |
| NaSal | 54-21-7 | 100 g | 25 | 5.59×10^-7^ |
| TEOS | 78-10-4 | 500 mL | 38 | 2.51×10^-6^ |
| Ethanol | 64-17-5 | 5 L | 140 | 4.93×10^-5^ |
| Methanol | 67-56-1 | 5 L | 90 | 1.35×10^-5^ |
| Hydrochloric acid | 7647-01-0 | 500 mL | 20 | 1.80×10^-6^ |
| MPTMS | 4420-74-0 | 25 g | 36 | 1.44×10^-5^ |
| Ammonium hydroxide solution | 1336-21-6 | 500 mL | 12 | 4.82×10^-7^ |
| CdSe/ZnS QDs | NA | 1 g | 5000 | 1.62×10^-2^ |
| Chloroform | 67-66-3 | 500 mL | 50 | 1.51×10^-4^ |
| 3-MPA | 107-96-0 | 25 g | 20 | 2.01×10^-6^ |
| DMSO | 67-68-5 | 500 mL | 72 | 5.06×10^-5^ |
| Anti-cTnI mAb | No.RC4T21-Y302 | 1 mg | 2700 | 0.549 |
| cTn complex Abs | No.4TC2-20C6cc | 1mg | 2100 | 0.427 |
| Recombinant anti-cTnI mAb | No.RC4T21-RecR85 | 1mg | 2700 | 0.549 |
| Troponin C cardiac | TnC,No.4T27cc-7B9cc | 1mg | 1650 | 0.335 |
| Sample pad | Glass fibre RB45 | 300×200 mm | 6/sheet | 7.07×10^-3^ |
| Conjugate pad | Glass fibre RB65 | 300×200 mm | 5.5/sheet | 1.78×10^-3^ |
| Nitrocellulose (NC) membrane | CN140 | 100 m×25 mm | 4000/roll | 0.124 |
| Absorbent pad | SX27 | 300×200 mm | 3/sheet | 5.36×10^-3^ |
| PVC backing card | SM31-40 | 300×80mm | 4.8/sheet | 4.12×10^-2^ |
| PBS | P4417 | PBS | 1069 | 4.49×10^-2^ |
| BSA | 9048-46-8 | 25 g | 208 | 8.99×10^-3^ |
| PEG1500 | 25322-68-3 | 500 g | 70.9 | 8.50×10^-6^ |
| Tween 20 | 9005-64-5 | 500 mL | 47 | 1.08×10^-2^ |
| Sucrose | 57-50-1 | 500 g | 27 | 4.92×10^-4^ |
| PVP24k | 9003-39-8 | 100 g | 82.9 | 4.97×10^-5^ |
|  |  |  |  | Total: 2.27 |

# References

Bai, T., Wang, M., Cao, M., Zhang, J., Zhang, K., Zhou, P., et al. (2018). Functionalized Au@Ag-Au nanoparticles as an optical and SERS dual probe for lateral flow sensing. *Anal. Bioanal. Chem.* 410, 2291–2303. doi: 10.1007/s00216-018-0850-z

Han, G.-R., Ki, H., and Kim, M.-G. (2020a). Automated, universal, and mass-producible paper-Based lateral flow biosensing platform for high-performance point-of-care testing. *ACS Appl. Mater. Interfaces* 12, 1885–1894. doi: 10.1021/acsami.9b17888

Han, G., Koo, H. J., Ki, H., and Kim, M. (2020b). Paper/Soluble Polymer Hybrid-Based Lateral Flow Biosensing Platform for High-Performance Point-of-Care Testing. *ACS Appl. Mater. Interfaces* 12, 34564−34575. doi: 10.1021/acsami.0c07893

Khlebtsov, B. N., Bratashov, D. N., Byzova, N. A., Dzantiev, B. B., and Khlebtsov, N. G. (2019). SERS-based lateral flow immunoassay of troponin I by using gap-enhanced Raman tags. *Nano Res.* 12, 413–420. doi: 10.1007/s12274-018-2232-4

Lee, K. W., Kim, K. R., Chun, H. J., Jeong, K. Y., Hong, D. K., Lee, K. N., et al. (2020). Time-resolved fluorescence resonance energy transfer-based lateral flow immunoassay using a raspberry-type europium particle and a single membrane for the detection of cardiac troponin I. *Biosens. Bioelectron.* 163, 112284. doi: https://doi.org/10.1016/j.bios.2020.112284

Li, Y., Yao, Y., Hua, Q., and Li, J. (2025). Quantitative and rapid lateral flow immunoassay for cardiac troponin I using dendritic mesoporous silica nanoparticles and gold nanoparticles. *Anal. Methods* 17, 698–707. doi: 10.1039/d4ay02060j
